# An industrially potent rhamnolipid-like biosurfactant produced from a novel oil-degrading bacterium, *Bacillus velezensis* S2†

**DOI:** 10.1039/d4ra02572e

**Published:** 2024-08-05

**Authors:** Shahnaz Sultana, Rokaia Sultana, Md. Abdullah Al-Mansur, Md. Ahedul Akbor, Nasrin Akter Bhuiyan, Shamim Ahmed, Sabina Yasmin, A. H. M. Shofiul Islam Molla Jamal

**Affiliations:** a Institute of National Analytical Research and Services (INARS), Bangladesh Council of Scientific and Industrial Research (BCSIR) New Elephant Road Dhaka 1205 Bangladesh nayeembcsir@gmail.com akborbcsir@gmail.com nasrinakter.nstu@gmail.com shamimbcsir@gmail.com sabinausha@yahoo.com shofiuljamal@yahoo.com; b School of Life and Environmental Sciences, University of Sydney Darlington NSW 2008 Australia ssul0164@uni.sydney.edu.au; c Medicinal Chemistry and Molecular Pharmacology Department, Purdue University West Lafayette Indiana-47907 USA rsultan@purdue.edu

## Abstract

Surfactants can reduce the interfacial surface tension between two immiscible liquids making them a desirable component for various industrial applications. However, the toxic nature of chemical surfactants brought immense attention towards biosurfactants. Being biodegradable, biosurfactants are eco-friendly and considered safer for different commercial uses. This study focused on the production of biosurfactant from an oil-degrading bacteria and its functional efficacy for prospective industrial applications. Here, a promising oil-tolerant strain, *Bacillus velezensis* S2 was isolated from oil contaminated sites which showed >50% degradation of convoluted crude oil within 28 days in comparison to a control. The isolate was then found to produce an excellent surface-active compound with an emulsification index of 67.30 ± 0.8% and could reduce the surface tension up to 36.86 ± 0.36 mN m^−1^. It also manifested a critical micelle concentration of 45 mg L^−1^ while reducing the surface tension from 72 to 30 mN m^−1^. When extracting biosurfactant from isolated bacteria, ethyl acetate extraction showed 1.5 times greater efficacy than chloroform : methanol extraction. The purified biosurfactant was characterized using TLC, ^1^H NMR, 13C NMR, FTIR, elemental analyses and spectrophotometric techniques leading to its identification as a rhamnolipid. The stability of produced biosurfactant at higher temperature (up to 180 °C) was determined by thermal analysis, endorsing its application in high temperature reservoir conditions. Additionally, the extracted biosurfactant showed excellent foaming efficacy with insignificant antibacterial and cytotoxic responses, which indicates their potential application in cleaning and cosmetics industries. Thus, the present study outlines a bi-functional novel isolate *Bacillus velezensis* S2 which could play a significant role in oil remediation from the environment as well as serve as a potential source of non-toxic and eco-friendly biosurfactants for various industrial applications.

## Abstract 2

An oil-degrading bacteria, *Bacillus velezensis* S2, was found to produce rhamnolipid-like biosurfactant which showed significant emulsification and foaming activity while reducing the surface tension remarkably. This offers more ecological sustainability than the toxic chemical surfactants.

## Graphical abstract

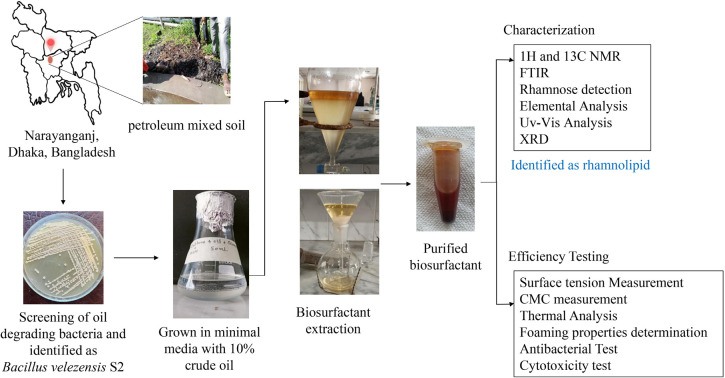

## 1. Introduction

Environmental pollution is a burning problem confronting the current world which occurs due to the high accumulation of hydrocarbons, chemicals, solvents, heavy metals *etc.* These pollutants have serious harmful effects on living organisms and also can hamper the economic growth in developing countries especially by polluting the aquatic environment.^1^ Crude oil being one of these hazardous pollutants, shows toxic and hydrophobic properties that hamper the diversified growth of the ecosystem.^2^ Moreover, crude oil spills in the environment undergo physico-chemical changes of properties resulting in the exposure of toxic polycyclic aromatic hydrocarbons (PAHs).^3^ Some lipophilic PAHs tend to spread through the food web and get bioaccumulated^3–5^ affecting the biodiversity of nature.^2^ A report in 2019 (ref. 6) stated that leakage of around 10 000 liters of heavy fuel oil into the Karnaphuli River of Bangladesh could spread over 16 kilometers and posed a threat to diversified aquatic lives including dolphins around the exposure site. However, chemical remediation of such crude oil contaminants from the surfaces is impeded by high cost and secondary pollution problems.^7^ Therefore, the progress in sustainable innovations has directed the quest for biological approaches to remediate crude oil from contaminated sites. In this regard, several microbial species such as *Pseudomonas*, *Bacillus* and *Streptomyces* have been reported for their capability to grow in oil contaminated stressful areas.^8–11^ These microbes have been reported to degrade heavy hydrocarbon moieties present in oil by producing active surfactants.^8,12^ The referred to biologically produced surfactants are known as biosurfactants which has led to the theory of eco-friendly surfactant production from natural sources.

Biosurfactants are amphipathic compounds having both hydrophilic and hydrophobic ends produced by living organisms.^13^ Due to such structural property, it can interact at the interface between aqueous and non-aqueous systems showing active surface activity. Glycolipids, lipopeptides or lipoproteins, neutral lipids, phospholipids, substituted fatty acids and lipopolysaccharides *etc.* are different forms of biosurfactant.^14,15^ The increased popularity held in the concept of biosurfactants is because of its numerous advantages over chemical surfactants. For instance, their low-toxicity, eco-friendly nature, higher biodegradability, natural stability at extreme conditions like pH, salinity and high or low temperature make them more valuable than the synthetic counterparts.^16,17^ As a consequence, these biomolecules have promising applications in multi-functional purposes such as oil recovery, cleaning, food processing, soil remediation, textiles industry, cosmetics, personal care industry and so on.^18,19^ Additionally, some biosurfactants conjugated with nano-particles (like Ag, Cu) or specialized amino acid have shown potential antibacterial activity against pathogens, proving their worth in this antibiotic retrospect era.^20,21^ Therefore, the microbes that can remove long-chained hydrocarbon containing waste oil from the environment and produce value added surfactants like biomolecules parallelly, are of great interest for sustainable ecosystem.

It is well known that oil-contaminated sites contain high volume of organic pollutants and theoretically the microbial diversity present there must degrade the organic contaminants to get energy for living. However, biosurfactant production has been proved as a major strategy of the microbes to break down the organic pollutants.^22,23^ The current study reports about such an efficient oil-degrading bacterial strain isolated from crude oil contaminated site which is also a source of potential biosurfactant compounds having prospective industrial uses.

## 2. Materials & methods

### 2.1 Materials

All media, chemicals, reagents and solvents used in the current study were of analytical grade; including: isooctane (C_8_H_18_, ≥ 99%, Sigma-Aldrich, USA), agar base (Himedia, India), nutrient agar (NA) (Scharlau, Spain), nutrient broth (Merck, USA), *n*-hexane (C_6_H_8_, ≥95%, Sigma-Aldrich, USA), acetone (C_3_H_6_O, ≥99%, Sigma-Aldrich, USA), chloroform (CHCl_3_, ≥99%, Sigma-Aldrich, USA), methanol (CH_3_OH, ≥99.8%, Sigma-Aldrich, USA), sulfuric acid (H_2_SO_4_, 95–97%, Scharlau, Spain), ethanol (C_2_H_5_OH, 99.8%, Sigma-Aldrich, USA), Mueller–Hinton agar (Himedia, India), agarose gel (Genedire, USA), dimethyl sulfoxide (DMSO, 99.99%, Sigma-Aldrich, USA), sodium chloride (NaCl, 99.9%, Sigma-Aldrich, USA), potassium chloride (KCl, Scharlau, Spain), potassium biphosphate (KH_2_PO_4_, Scharlau, Spain), disodium phosphate (Na_2_HPO_4_, Scharlau, Spain), dipotassium phosphate (K_2_HPO_4,_ Scharlau, Spain), magnesium sulfate heptahydrate (MgSO_4_·7H_2_O, Sigma-Aldrich, USA), calcium chloride (CaCl_2,_ ≥97%, Sigma-Aldrich, USA), NaOH (Sigma-Aldrich, USA), tween-20 (Scharlau, Spain), tween-80 (Pure Pharma, GmbH, Germany), and sodium dodecyl sulfate (SDS, Pure Pharma, GmbH, Germany).

### 2.2 Sample collection and screening of crude oil degrading bacteria

Three types of samples (40 years old oil contaminated soil, 15 years old oil contaminated soil and waste oil) were collected from MEGHNA Fatullah Depot, Narayanganj (23′33′–23′57′N and 90′26′–90′45′ E), Bangladesh. The samples were serially diluted (up to 10^−7^) in phosphate-buffered saline (137 mmol per L NaCl, 10 mmol per L H_3_PO_4_, and 2.70 mmol per L KCl, pH 7.4). Then 0.1 mL from 10^−3^, 10^−4^ and 10^−5^ dilutions were spread on to Nutrient Agar (NA) and the plates were incubated at 37 °C for 24–48 h. After that, a single distinct colony was surface streaked on the same culture media under the previous conditions, to obtain isolated colonies.

Bacterial tolerance to petroleum crude oil was examined by incubating the isolated colonies in minimal salt medium (MSM) broth constituting of 2.94 mM KH_2_PO_4_, 7.01 mM K_2_HPO_4_, 0.81 mM MgSO4·7H_2_O, 0.18 mM CaCl_2_, and 1.71 mM NaCl^24^ and supplemented with 1% crude oil for 7 days, at 180 rpm and 30 °C. Each distinct oil tolerant cultures was further investigated for their oil degradability and survivability by determining their growth on MSM supplemented with crude oil at different concentrations (2%, 5%, 10%, 20%, 40%, 50%, 75%, and 100%; v/v).^25^ 1 mL of culture solution (OD_600_ = 0.32) was transferred to each treatment. After incubating them under the same condition, the viable bacterial count was determined on nutrient agar media through spread plate technique on the following day.

### 2.3 Isolate characterization and identification

#### 2.3.1 Phenotypic and biochemical characterization

As a potential oil degrader, the target isolate (ODB-2) was initially characterized based on colony morphology on NA media and microscopic appearance (Zeiss Primo Star Microscope, Germany). The biochemical characterization was performed according to the procedure described in bergey's manual of determinative bacteriology.^26^

#### 2.3.2 Phylogenetic analysis by 16S rRNA gene sequences

Total genomic DNA was extracted from selected isolates to identify based on 16S rRNA gene sequence analysis using boil template method^27^ (ESI File†).

### 2.4 Degradation of crude oil by the most potent isolate

The potential oil degrading isolate was then tested for its oil degrading capability employing gravimetric analysis, spectrophotometric analysis, and gas chromatography (GC) analysis. For this, the isolate was inoculated in 100 mL MSM contained with 10% of (v/v) (∼81 g) crude oil as sole carbon source, followed by the incubation at 30 °C and 120 rpm for 28 days. Un-inoculated flask having the identical system was prepared as controls, so that the abiotic losses of crude oil, particularly for evaporation could be determined by the un-inoculated control. The records for each treatment were taken in triplicates and calculated in reference to un-inoculated controls.

#### 2.4.1 Gravimetric analysis of crude oil biodegradation

The quantitative degradation of crude oil was initially determined by gravimetric method^28^ (described in ESI File†).

#### 2.4.2 Spectrophotometric method

The quantitative analysis of oil biodegradation by UV spectrophotometry was determined according to the standard protocol described by Liu *et al.*^29^ with few modifications (described in ESI File†).

#### 2.4.3 Oil degradation analysis by gas chromatography (GC)

Residual crude oil samples extracted after 7 days of incubation was analyzed by GC to confirm the degradation efficacy of the strain in reference to control condition^30^ (described in ESI File†).

### 2.5 Determination of biosurfactant producing ability

The qualitative studies that are frequently used as quick and simple experiments to check the presence of any surface-active compound in a solution were performed in this study which includes oil spreading test, drops collapse assay, BATH test, emulsification index, and most importantly, surface tension measurement.^31,32^

#### 2.5.1 Oil spreading assay (OSA)

OSA test was performed according to the method described by ref. 32 to determine the presence of biosurfactant in culture solution (described in ESI File†).

#### 2.5.2 Drop collapse test (DCT)

DCT is a qualitative test for screening of biosurfactant production and this test was performed according to Bodour and Miller-Maier^31^ (described in ESI File†).

#### 2.5.3 Emulsification index (E_24_)

The emulsification index (E_24_) test was performed to evaluate the emulsifying ability of culture supernatant as reported by ref. 33 (described in ESI File†).

#### 2.5.4 Bacterial adhesion to hydrocarbon (BATH) assay

The relative hydrophobicity of bacterial cells was measured by a BATH assay described by ref. 34 (described in ESI File†).

#### 2.5.5 Surface tension (ST) determination

The ST of the biosurfactant-containing CFS was determined according to the Wilhelmy plate method^35^ using Ossila Contact Angle Goniometer (L2004A1, Netherland) equipped with a high-resolution video-camera system and dedicated computer software (described in ESI File†).

### 2.6 Biosurfactant extraction

Crude biosurfactant was extracted following the acid precipitation and liquid–liquid solvent extraction method explained by ref. 36 (described in ESI File†).

#### 2.6.1 Dry weight measurement of biosurfactants

Two sterile Petri plates were weighed and the unpurified biosurfactant produce from chloroform : methanol liquid–liquid extraction and ethyl acetate mediated extraction were poured onto the plates separately. After drying them for 2 hours at 80 °C on the hot air oven, the plates were weighed again.^37^ The dry weight of the biosurfactants was calculated as follows:Dry weight of biosurfactants = (weight of the dried biosurfactant containing plate − weight of the empty plate)

#### 2.6.2 Microscopic analysis

To examine the microstructure of the extracted biosurfactant, the product was gently stirred to ensure a homogenous sample. A loop-full biosurfactant was spread on a glass slide of 15 × 20 mm^2^, previously cleaned with 70% ethanol and dried properly. A 0.17 mm thick coverslip was placed on top to the spread sample for ensuring zero air gaps between the sample and the coverslip. An optic microscopy was carried out using a four-objective binocular microscope (Zeiss Primo Star Microscope, Germany). The sample was observed under 10×, 40× and 100× lens at room temperature (24 °C).

### 2.7 Purification of biosurfactant and thin layer chromatography

To analyze the compound structure, crude biosurfactant was purified using the modified method of ref. 38 and 39 where the precipitate or biosurfactant was allowed to run through open column chromatography using a laboratory burette (ESI File†).

### 2.8 Structural characterization of obtained biosurfactant product

#### 2.8.1 Nuclear magnetic resonance (NMR) analysis

Isolated pure compounds were studied under nuclear magnetic resonance (NMR) spectrometer (Bruker 400 NMR spectrometer) for further structural elucidation with a unity of 400 MHz. During the measurement of 1H spectra, the instrumental conditions were recorded as; frequency (399.73 MHz), spectral width (6410.3 Hz), acquisition time (2.556 s), relaxation delay (1 s), pulse angle flip (90°) and number of transients.^40^ The instrumental conditions for carbon spectra were: frequency (100.51 MHz), spectral width (25 510.2 Hz), acquisition time (1.285 s), relaxation delay (7 s), pulse angle flip (90°) and number of transients (1000). Here, deuterated chloroform, CDCl_3_ was used to dissolve the compounds. The structures of the compounds were elucidated by spectroscopic analysis as well as by comparing with previously published NMR data. The chemical shifts are expressed in ppm.

#### 2.8.2 Fourier transform infrared spectroscopy (FTIR) analysis

Fourier-transform infrared (FTIR) spectra of the purified biosurfactant were recorded to identify the functional groups and chemical nature of the product. For this, the sample was dried and converted in powdered form. The powdered sample was mixed with KBr and FT-IR spectrum was recorded over the range 450–4000 cm^−1^ using FTIR Spectrometer (Shimadzu, FTIR-8900/8400S Japan). Attenuated Total Reflection (ATR)-FTIR spectra (Model-IR Prestige 21, Shimadzu, JAPAN) of the sample were also taken with the diamond crystal to get complete information of all functional groups of the sample. The spectrum was recorded in the range of 4000 cm^−1^ to 650 cm^−1^ and the entire scans were 30 in number, with a spectral resolution of 4 cm^−1^.^41^ Before analysis of each sample, the instrument was standardized, and the ATR diamond crystal was cleaned using 2-propanol (70%).

#### 2.8.3 Rhamnose detection

The UV-spectrophotometer (UV-2600, SHIMADZU, Japan) was used to determine the presence of carbohydrate groups in the biosurfactant molecule by rhamnose test using the method of ref. 42 (ESI File†).

#### 2.8.4 Elemental analysis

The elemental contents (C, H, O, N, and S) of the biosurfactant samples (before and after purification) were measured by an elemental analyzer (CHNS analyzer vario MICRO cube Elementar, Germany).

#### 2.8.5 UV

The ultraviolet-visible (UV-vis) absorption spectra of purified biosurfactant sample were taken using an UV-vis-NIR (near-infrared) spectrophotometer (Shimadzu UV 2600 ISR Plus) between 200 and 800 nm wavelength region to study the optical response of sample.

#### 2.8.6 X-ray diffraction (XRD) analysis

Extracted biosurfactant was studied with X-ray diffraction (XRD) experiments (SmartLab SE, Rigaku, Japan), with the aim to describe their structural molecular organization at few Angstrom resolutions. The data had been recorded as a function of temperature by fixing scanning range, 2*θ* = 5° to 90° and step width of 0.01°, Here, a copper tube made of ceramic (CuKα, *λ* = 1.54060 Å) was chosen as the X-ray source with a step scan of 15° min^−1^ and the voltage and current were fixed at 40 kV and 50 mA, respectively.

#### 2.8.7 Thermal properties analyses

The thermal properties of the extracted biosurfactant were performed by a simultaneous thermal analyzer (Model: STA449F3, NETZSCH, Weimar, Germany) (described in ESI File†).

#### 2.8.8 Determination of foaming property

The purified biosurfactant was tested for its foaming abilities following the method described by ref. 43 (ESI File†).

#### 2.8.9 Determination of critical micelle concentration (CMC)

In this study, the CMC of purified biosurfactant was determined by measuring its surface tensions for a series of concentrations (0–100 mg L^−1^).^44^ In general, the ST of two immiscible surfaces will drop down with the increased addition of surfactant up to a certain concentration. However, once the saturation point is reached, the ST would become independent of surfactant amount and will remain unchanged. The surfactant concentration of this saturation point is known as CMC beyond which the molecules get aggregated and start to form micelles. Once the micelles formation is achieved, topping up the surfactant amount in the interface would be worthless.^36^

#### 2.8.10 Antibacterial test

To determine if the produced biosurfactant had any antibacterial property, *Staphylococcus aureus* (ATCC 14458) and *Escherichia coli* O157:H17 (ATCC 43895) were chosen as Gram-positive and Gram-negative representative. For this, agar well diffusion assay was performed according to the technique recommended by ref. 45 and 46 (ESI File†).

#### 2.8.11 Cell cytotoxicity

The cytotoxic response of purified biosurfactant was investigated using the Trypan Blue Exclusion method^47^ (described in ESI File†).

### 2.9 Statistical analysis of experiments

Around all the experiments, particularly the data for bacterial growth, biochemical parameters, and functional screening like oil displacement, emulsification, surface tension, foam test was performed in three replicates. The statistical package SPSS (version 26.00, Chicago, IL, USA) was used for data processing with a two-way ANOVA analysis of variance. Duncan's multiple range examination was applied at a 5% probability level to determine significant disparities in the mean values.

## 3. Results and discussion

### 3.1 Isolation and screening for oil degrading bacteria

**Fig. 1:**
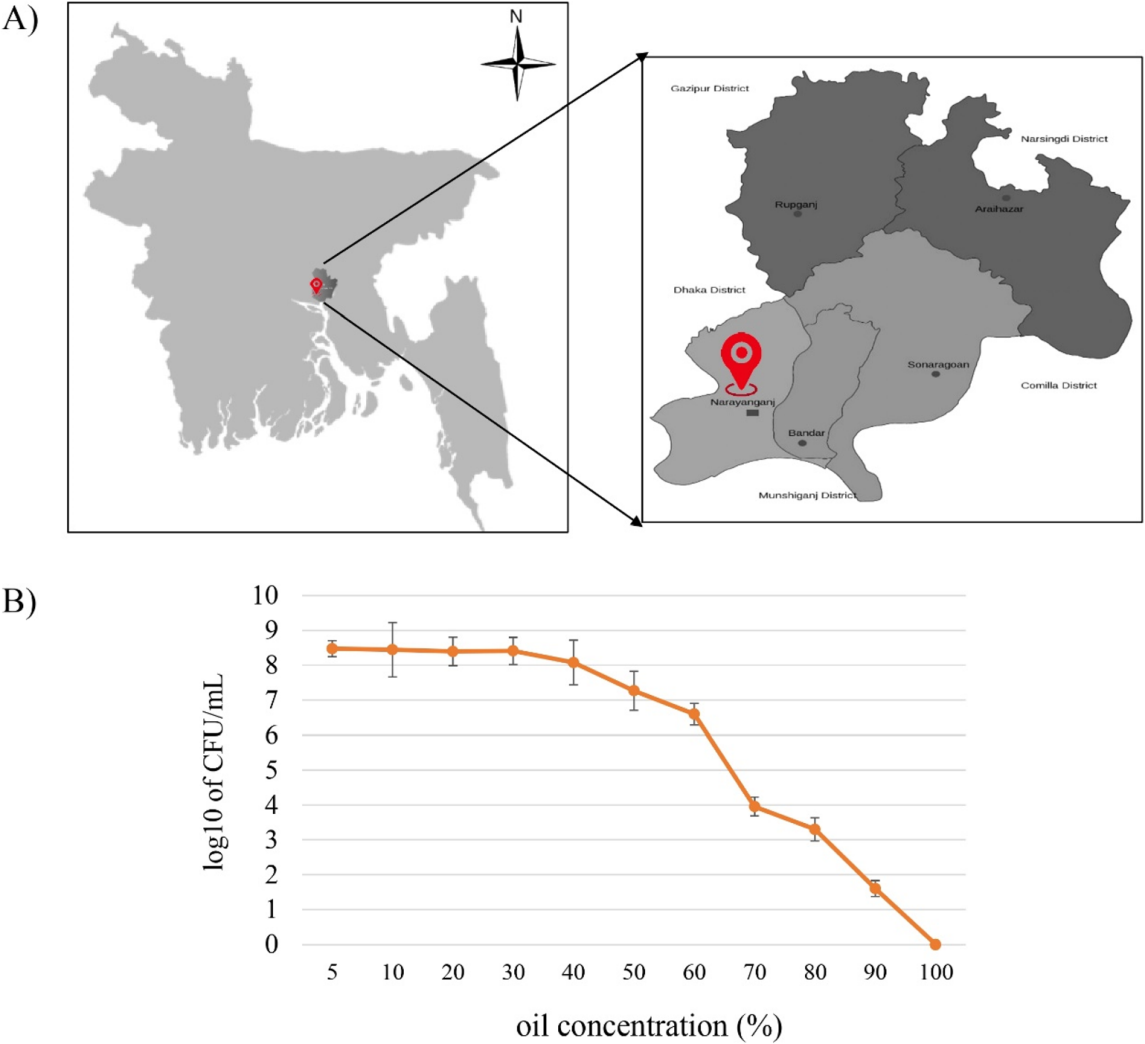
A representative illustration of Bangladesh map with a magnified view of sampling site (A); a line graph showing the oil tolerance capability of isolate ODB-2 at different crude oil concentration based on its viability upon 24 hours of incubation (B). The experiment was done in triplicate where the bars represent standard deviation (±SD).

Due to the intriguing and eco-friendly properties of biosurfactants, an emerging interest and active research are extending the concept to incorporate such compounds in potential industrial applications.^48–51^ Moreover, biosurfactants derived from oil degrading bacteria are gaining strong status for both restoring environmental conditions as well as prospective commercial uses.^52–55^ This study made use of potential oil degrading bacteria (ODB), isolated from oil contaminated soil samples collected from oil contaminated soil (15 years and 40 years old) and waste oil samples collected from MEGHNA Fatullah Depot, Narayanganj (23′33′–23′57′N and 90′26′–90′45′E), Bangladesh (Fig. 1A) for their application in petroleum remediation capability and production of industrially valuable biosurfactant. Initially, a total of 16 distinct bacterial colonies were isolated among which, 7 isolates could grow in presence of 1% crude oil containing MSM media. The isolates were labelled as oil degrading bacteria (ODB) 1 to 7. To harness the possible oil degradation ability of the 7 isolates, they were tested for scoring their oil tolerance capability, most of them were found to be tolerant to oil concentrated from 2% to 40% while only one isolate could tolerate up to 90% of crude oil contained in batch culture system (Fig. 1B). This isolate (ODB-2) was selected as a potential oil degrader for further study.

#### 3.1.1 Identification of potential oil degrader

**Fig. 2:**
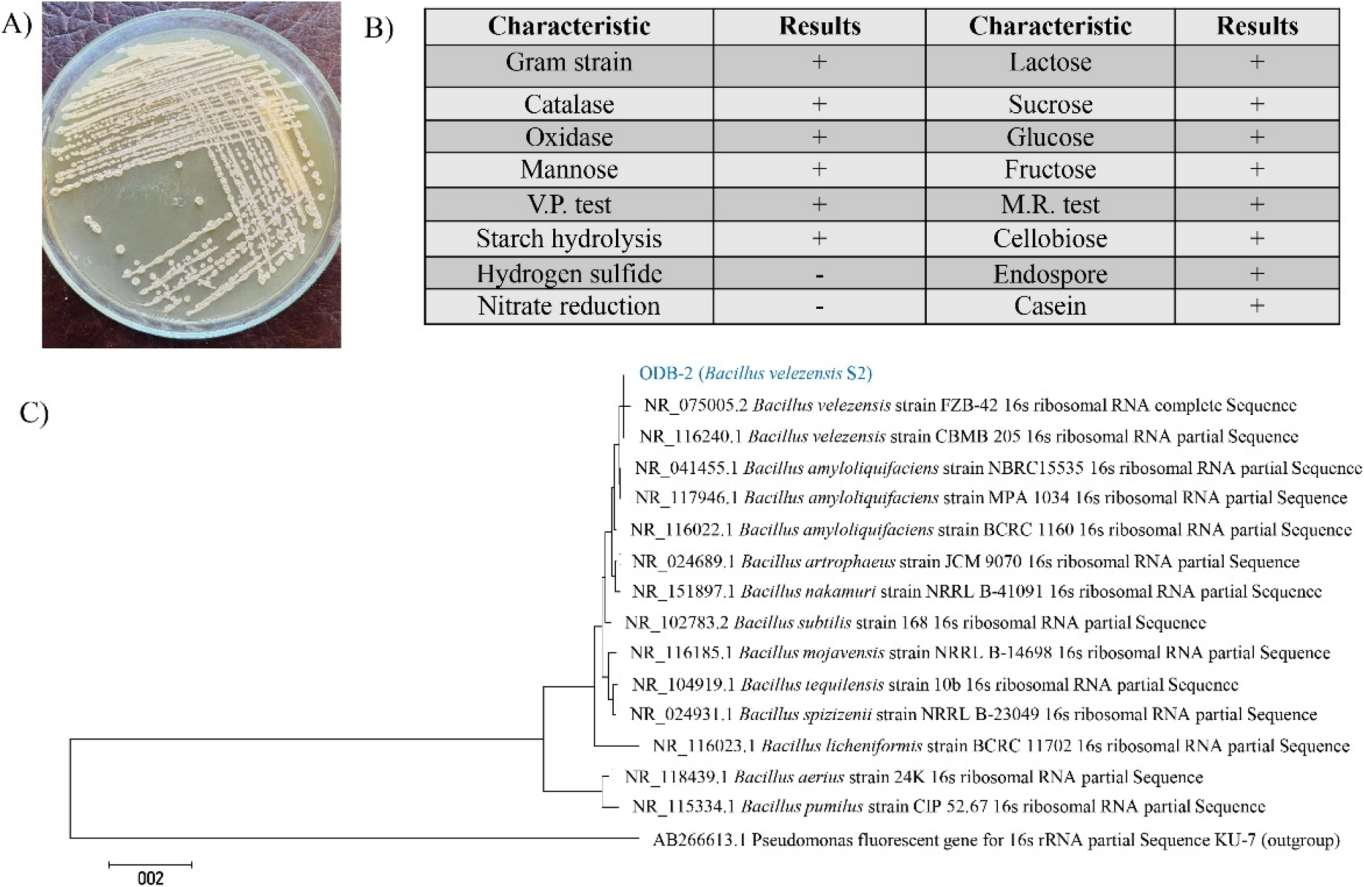
Characterization and identification of potential oil biodegrader, ODB-2 as *Bacillus velezensis* S2. Colony morphology on NA plate (A), biochemical characteristics, where “+” indicates positive response; “−” indicates negative response (B), phylogenetic tree of 16s rRNA of ODB-2 showing its relation to the available sequence on the NCBI database (C).

Morphological, microscopic, and biochemical characteristics of the isolate ODB-2 showed its resemblance with *Bacillus* forming an irregular, moderate sized, white, skin-like pellicles with mucoid texture and a ridged surface when grown in NA (Fig. 2A). When cultured at 37 °C for 24 h in NA medium, the culture was found as Gram-positive, rod shaped, spore containing strain under microscope. The biochemical analysis also revealed the isolate as *Bacillus* strain with positive catalase test, Methyl red and Voges Proskauer (MR–VP) test, different sugar test, oxidase test, and negative hydrogen sulfide and nitrate reduction test (Fig. 2B). The isolate was then identified as *Bacillus velezensis* S2 based on 16 s rRNA partial gene sequence that showed 99.86% similarity to *B. velezensis* strains CBMB205 (GenBank accession number is NR_116240.1).

The 16S rRNA gene partial sequence of strain S2 was submitted to the NCBI GenBank database under accession no. gb |OR337901|. The phylogenetic tree of the 16S rDNA of S2 strain and its relation to the available sequences on the NCBI GenBank database is illustrated in Fig. 2C.

Interestingly, different *B. velezensis* isolates were reported to contain the genes responsible for the degradation of lignocellulosic, cellulosic and hemicellulosic materials.^56^ In other studies, *B. velezensis* strains were also investigated for their ability to utilize various types of agro-industrial byproducts in biofuel production.^57^ Some experiments also reported it as a probiotic, bile tolerant, riboflavin producer and exopolysaccharide (EPS) synthesizer^58^ while other reported this spp as lipid producer.^59^ Meena *et al.*, on the other hand, found a *Bacillus velezensis* strain showing both surfactant producing and crude oil degrading ability.^60^ Overall, this spp appears to be a great interest of study while thinking of industrially valuable biocompounds.

### 3.2 Crude oil biodegradation

**Fig. 3:**
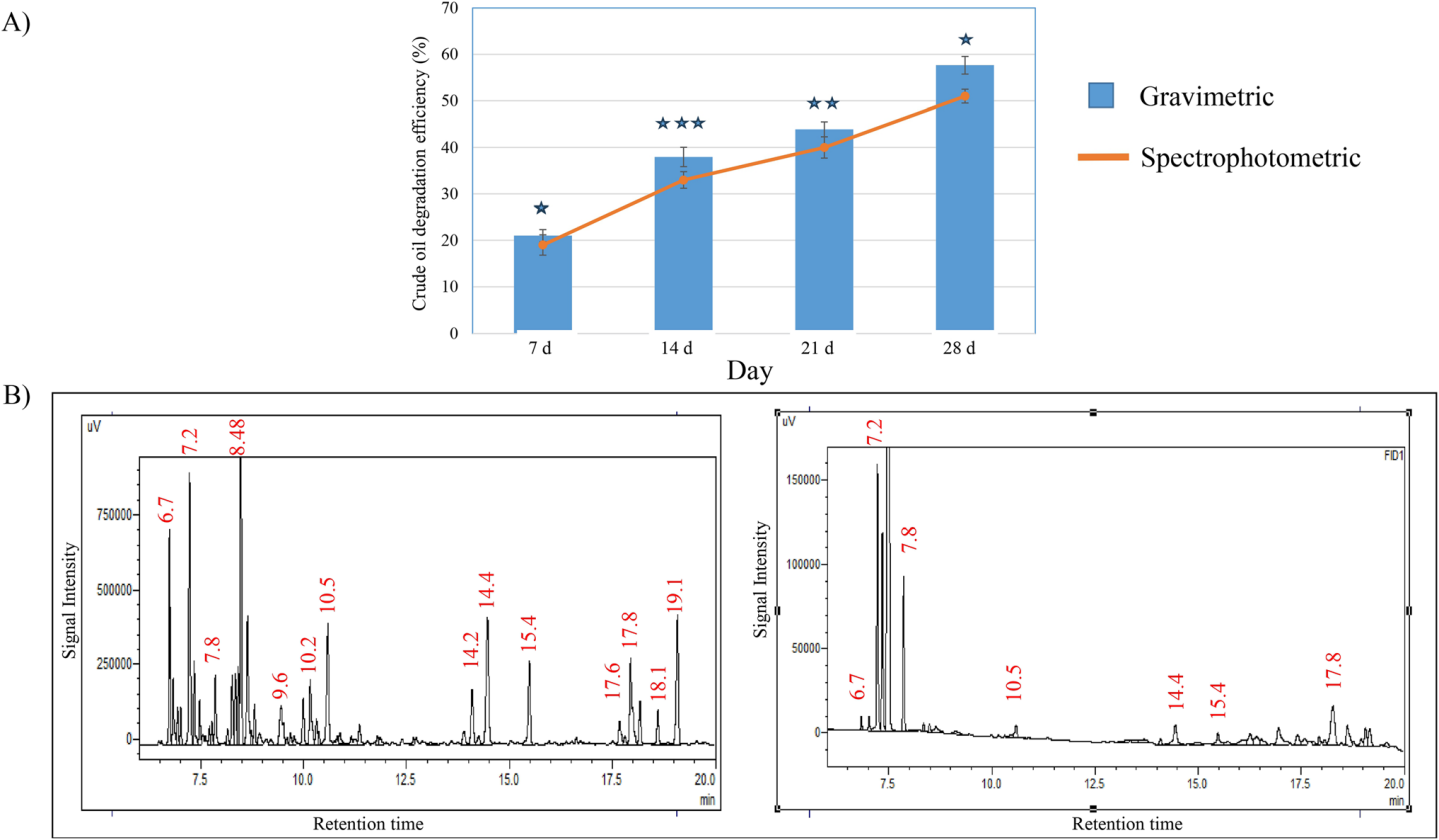
An outline describing the oil biodegradation ability of *B. velezensis* S2. The bar diagram depicts the quantitative analysis of crude oil degradation by gravimetric method while the line graph demonstrates the same by spectrophotometric assay (A). The GC chromatogram (left side) represents the alkane fractions present in the untreated crude oil (control), while the right-side image clearly shows the reduced alkane fraction of crude oil treated with S2 after 1 week of incubation (B). The statistical analysis for the gravimetric and spectrophotometric analysis was performed using SPSS statistical package (version22) for the one-way ANOVA, where the values were found as significant shown by asterisk (*, *p* < 0.05; **, *p* < 0.01; and ***, *p* < 0.001).

When the potential isolate S2 was tested for its oil degradation efficiency, the gravimetric analysis showed more than 50% degradation of crude oil in reference to control (un-inoculated) following 28 days of incubation period (Fig. 3A). Similar pattern of oil degradation rates was proved by the spectrophotometric experimental method, where the degradation rates were found as 19.13%, 33.09%, 40.47% and 51.87% at the incubation intervals of 7, 14, 21 and 28 days respectively (Fig. 3A).

The GC-ECD chromatogram of oil biodegradation was recorded after 1 week of the culture incubation (treated) and was compared to that of the abiotic control sample (untreated crude oil containing MSM) (Fig. 3B). The signal intensities for various crude oil fractions in S2 treated culture were disappeared or significantly reduced by more than 5 folds indicating the efficiency of this strain in biodegradation and/or biotransformation of crude oil. This experiment was repeated for 3 times which demonstrated almost similar reduction of crude oil fraction.

An alike study showed that a consortium of six Gram-negative oil-tolerant bacteria could degrade about 42% of crude oil in 36 days which continued to the reduction of 83.70% of the oil in 75 days.^28^ Another study signified the efficiency of oil-tolerant bacterial consortium where it observed 50.63–55.43% of crude oil removal in 7 days supporting the use of consortium in facilitating remediation of oil-contaminated sites.^61^ Therefore, alone or with other, the isolate from this study may prove to be significant in biodegradation of crude oil.

### 3.3 Determination of biosurfactant producing ability

While considering different experiments to determine the producing capability of biosurfactant by the isolate, we chose the most common, rapid, and sensitive methods to be carried out. Among them, the OSA is considered as a simple and rapid biosurfactant detection method requiring a small volume of sample.^37^ In our OSA experiment, 1 drop of the culture CFS could produce 3.93 (±0.15) mm of clear zone (in diameter) by reducing the interfacial surface tension between water and crude oil. Similarly, the findings of Nayarisseri *et al.*^62^ reported two biosurfactant producers, where *Staphylococcus* and *Bacillus* showed positive response with a clearance zone of 1.8 mm and 2.5 mm respectively. As several studies proved that oil spreading area in this technique is directly related to the surface-active agent present in the solution,^63^ this finding proves the production of biosurfactant by S2 isolate.

Another sensitive test, the drop collapse assay endorsed the findings of OSA producing a flat/collapsed drop 58 s upon the addition of bacterial CFS on the top of equilibrated crude oil surface in microtiter plate. This occurred due to the surface tension reduction in the interface of two immiscible liquids.^64^ The record showed similarities with a separate study where biosurfactant producing *Bacillus* spp could collapse the drop within 58 s whereas *Staphylococcus* spp could do the same within 1 min 56 s.^62^

The emulsification index assay is an effective way to determine the presence of biosurfactant. It works on a concept that a culture grown in a media containing hydrophobic natured hydrocarbons (such as crude oil) would emulsify the present hydrocarbons^40^ if the culture can produce any surfactant like compound. In this study, the CFS from S2 culture showed an emulsification index of 67.30 ± 0.8%, whereas it is reported that E_24_ greater than 30% can be indicated as higher activity of surfactant.^62^ Accordingly, when *Pseudomonas aeruginosa* showed an emulsification activity of (55.56–60)% in a study,^40^ it was reported as a biosurfactant producer. A likely report also exhibited that *P. guguanensis* strain extracted product showed an E_24_% of 52 ± 0.33% (ref. 65) and was proved to be a biosurfactant producer.

Another technique to screen out biosurfactant producing bacteria is BATH test which can determine surfactant producer by identifying their cell hydrophobicity in present of crude oil.^62^ Here, cell hydrophobicity can be indicated by the adherence or affinity of bacterial cells to the crude oil portion in the batch culture system. In this experiment, the cell adherence for S2 strain was found as (92.44 ± 0.65)% which can be regarded as a positive biosurfactant producer following a similar study conducted by.^62^

**Fig. 4:**
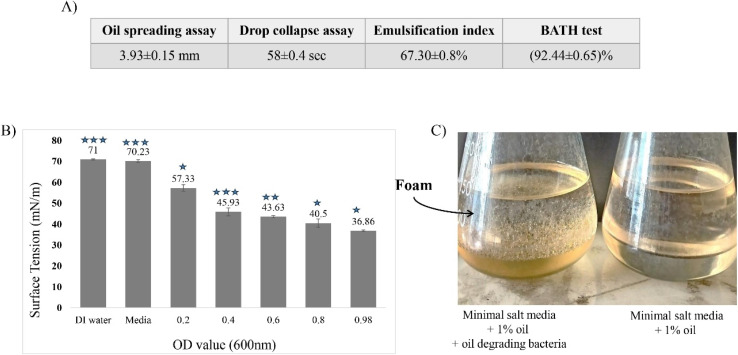
A diagram exhibiting the experiment outcomes of biosurfactant production by S2 strain. Here, the table shows positive indications of biosurfactant production in terms of OSA, drop collapse assay, emulsification index and BATH test (A); a gradual reduction of surface tension in S2 treated media with the increasing cell density compared to untreated conditions (DI water and only media) (B); a picture showing the crude oil containing media appearances in terms of treated (with S2) and untreated (without S2) conditions (C). The statistical analysis was performed using SPSS statistical package (version22) for the one-way ANOVA, where the values were found as significant shown by asterisk (*, *p* < 0.05; **, *p* < 0.01; and ***, *p* < 0.001).

The reduction in surface tension is another strong indication of the presence of biosurfactant in a solution.^66^ In this experiment, the surface tension measurement of CFS showed a gradual reduction in surface tension with the increasing cell number. As shown in Fig. 4B, *B. velezensis* S2 decreased the surface tension by (36.86 ± 0.36) mN m^−1^ having an OD_600_ value of 0.98 comparing to the control indicating the existence of surfactant like compound in the culture. Similarly, other reports on *P. aeruginosa* IITR48,^67^*Staphylococcus* and *Bacillus* spp;^62^ which were found as biosurfactant producer, could reduce the surface tension by 29 N m^−1^, 42 mN m^−1^ and 38 mN m^−1^ respectively. Reports claim that an efficient biosurfactant can reduce the surface tension of pure water from 72 mN m^−1^ to 30 mN m^−1^.^54^ For instance, 10 μM surfactin could reduce the surface tension of water (72 mN m^−1^)to 27 mN m^−1^ (ref. 68) while rhamnolipids, sophorolipids, and trehalose were reported to reduce the surface tension to <30–33 mN m^−1^.^69–71^

As we have used MSM media for the batch culture system, no surface-active compound could be present in the CFS unless they were produced by the culture. In this study, a direct correlation is visible in OSA, drop collapse, emulsification index, BATH test and surface tension assays. Similar correlation was reported by Bodour and Miller-Maier^31^ between drop collapse method and surface tension while they were determining the production of biosurfactant.

### 3.4 Crude biosurfactant extraction

**Fig. 5:**
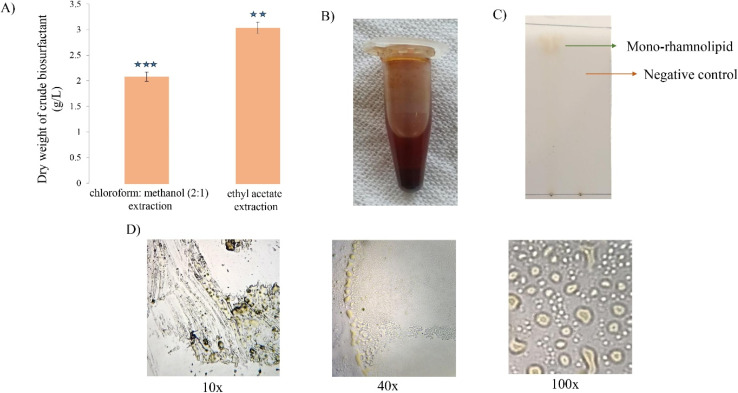
Comparison of biosurfactant amount (g L^−1^) extracted from chloroform : methanol (1 : 1) and ethyl acetate treatment (A); crude biosurfactant (B); TLC performed on purified biosurfactant where the left lane contains sample along with ethyl acetate and the right lane contains ethyl acetate only (C); biosurfactant molecules under compound microscope at 10×, 40× and 100× resolution (D). The statistical analysis was performed using SPSS statistical package (version22) for the one-way ANOVA, where the values were recorded as significant shown by asterisk (**, *p* < 0.01; and ***, *p* < 0.001).

In this study, ethyl acetate was found as a good recovery solvent for biosurfactant extraction compared to chloroform : methanol (2 : 1) giving the yield quantity of 3.12 ± 0.11 g L^−1^ and 2.15 ± 0.09 g L^−1^ respectively. Hence, the dry weight of biosurfactant produced from ethyl acetate extraction weighed almost 1.5 times higher compared to the chloroform : methanol extraction. The impurified biosurfactant was brown in color with thick consistency. These results are in concordance with those found in previous works for biosurfactant reported by Patowary and his colleagues where ethyl acetate extracted biosurfactant from *P. aeruginosa*, resulted in 2.26 g L^−1^ honey brown crude biosurfactant.^72^ In opposed to that, the recovery outcome from this study is contradictory to the study of ref. 73 and 74 where ethyl acetate could recover least biosurfactant upon extraction. However, the European Union (EU) regulation recommends the use of ethyl acetate for solvent extraction process^74^ in the case of food applications and other human consumables. The microscopic analysis of biosurfactant showed oily droplet like composition under the lens (Fig. 5).

### 3.5 Structural characterization of obtained biosurfactant product

#### 3.5.1 Biosurfactant purification and TLC

Following biosurfactant purification, the fraction eluted after column chromatography using ethyl acetate (100%) showed only one spot on the TLC plates with *R*_f_ value 0.81 (Fig. 5C). Ghazi *et al.* reported the production of monorhamnolipid by *Pseudomonas guguanensis* with an *R*_f_ value of 0.83.^65^ A likely findings were revealed by Sun and his colleagues where a glycolipid produced from *P. aeruginosa* formed two spots of di and monorhamnolipid with *R*_f_ values 0.53 and 0.85, respectively on TLC analysis.^75^

#### 3.5.2 NMR

**Fig. 6:**
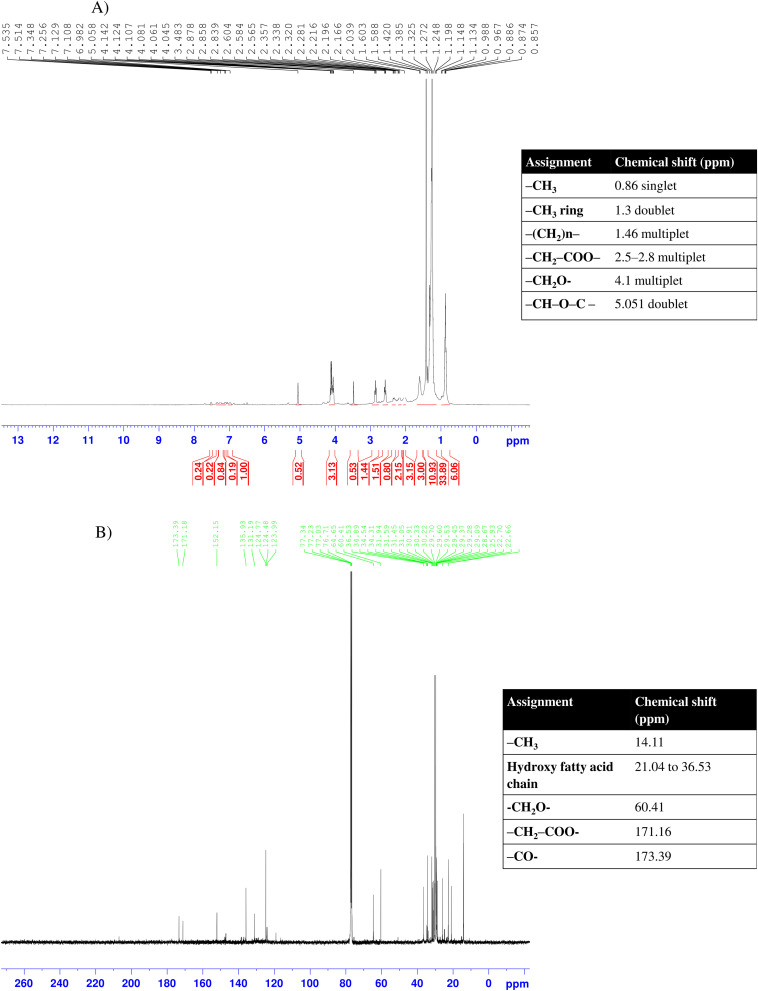
1H NMR spectrum (A), and 13C NMR spectrum (B) of purified biosurfactant extracted from *B. velezensis*.

The 1H and 13C NMR spectra of purified biosurfactant are illustrated in Fig. 6 showing some characteristic chemical shifts clearly indicative for rhamnolipid like biosurfactant.^76^ The long aliphatic chain and sugar ring were confirmed by the significant signals in the region of 0.86–1.5 ppm in 1H NMR analysis. Particularly the methyl rhamnosyl moiety is attributed to the doublet chemical shifts at around 1.3 ppm. The resonance peaks between 2.5 and 2.9 ppm indicated the probable occurrence of –COO–CH_2_– whereas the peak at 4.043 ppm denotes the existence of –CH_2_O– due to the β hydroxyl fatty acid chain in the sample. The doublet chemical shift of 1H NMR at 5.051 ppm confirmed the presence of –CH–O–C moiety in the sugar ring. Similarly, 13C NMR spectrum shows significant signals at 14.11 ppm and from 21.04 to 36.53 ppm denoting –CH_3_ and the presence of lipid in the sample respectively. Also, the signal for –CH_2_O– was generated at 60.41 ppm whereas the ester and ketonic group (C=O) peaks were shifted at 171.16 and 173.39 ppm respectively. The absence of any resonance peak for aromatic or amine group and the occurrence of long aliphatic chain with ketone and ester groups suggests the sample having chemical structure of mono-lipid rhamnolipid.^77–79^

#### 3.5.3 FT-IR analysis

**Fig. 7:**
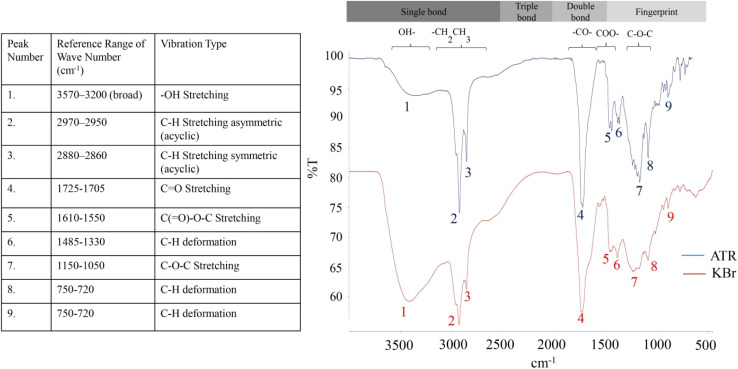
FT-IR spectrum of purified biosurfactant.

Fourier transform infrared (FT-IR) spectra of purified biosurfactant demonstrating maximum absorption at distinct wavenumber positions due to the presence of specific functional groups are shown in Fig. 7. Both KBr and ATR-FTIR spectrum showed several significant peaks at almost same range of wavenumber. The characteristic peak noticed at 3358 cm^−1^ in ATR-FTIR and 3406 cm^−1^ in KBr-FTIR confirms the stretching vibration of hydroxyl (–OH) functional group.^80,81^ The two consecutive peaks at the positions of 2927 and 2855 cm^−1^ (ATR-FTIR) are attributed to the stretching bands of aliphatic chains (–CH_2_– and –CH_3_ groups). The ketonic (C=O) group was identified by the sharply pointed band at wavenumber 1719 cm^−1^ and 1729 cm^−1^ in ATR and KBr spectrum respectively.^82^ The ester carbonyl group (COOR–) and the epoxy group (C–O–C) were confirmed by the stretching bands originated at 1552 and 1163 cm^−1^ respectively (ATR spectrum). In the fingerprint region (by the bands below 1500 cm^−1^ to 500 cm^−1^), the absorption bands between 1485 and 1330 cm^−1^ indicates the deformation vibrations of alkyl groups according to. All these peaks obtained from FT-IR analysis suggest the structure of biosurfactant as rhamnolipid homologue following the findings consistent with the other reported works.^83^

#### 3.5.4 Rhamnose test

**Fig. 8:**
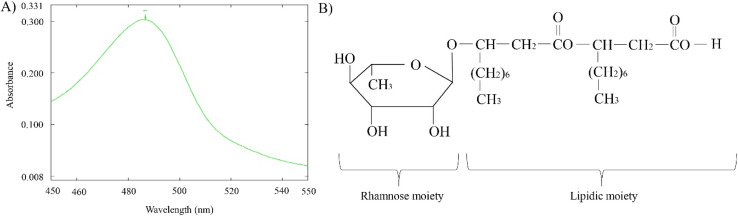
A diagram showing the presence of rhamnose moiety in the extracted biosurfactant structure; spectrophotometric analysis exhibiting the rhamnose positive test (A); the structure of monorhamnolipid indicating its rhamnose and lipid moiety in the structure (B).

Following the data derived from NMR and FTIR, a UV-vis spectrum based rhamnose test was performed for the purified biosurfactant and the results was found as positive (Fig. 8A) exhibiting a significant peak at 490 nm. This indicates the glucose moiety in the biosurfactant to be a rhamnose sugar.

All the findings of NMR, FTIR and rhamnose test suggested a likely structure of the compound having rhamnose and lipidic group. Compared to previous reports, the extracted biosurfactant which were confirmed as rhamnolipid showed similar experiment outcome.^76,77^ To date, *Pseudomonas* spp have been majorly reported to produce rhamnolipid like biosurfactant while *Bacillus* spp have been found as the potential secreter of surfactin-type biosurfactant.^84^ In fact, *Bacillus velezensis* have also been globally recorded as surfactin producer so far.^60,85^ To our best knowledge, this is the first time the isolated *B. velezensis* S2 strain from this study has been proved to synthesize rhamnolipid-like biosurfactant.

Rhamnolipid belongs to amphiphilic glycolipid group consisting of one or two rhamnose units (mono or di-rhamno) and attached to one or two lipid chains (mono or di-lipid). The variation in sugar moiety and the fatty acid chain quantity generates quite a number of rhamnolipid homologue in nature.^76,77^ Interestingly, the di lipid-rhamnolipids form an ester bond linking two fatty acid groups of the lipid chains. This ester group can transform to its stereoselective forms like ether, amide, hydrocarbon or ketone; opening a broad spectrum of biological functions compared to other biomolecules.^77–79^ This may explain the reason why some rhamnolipids are well known for their anti-bacterial or anticancer activity, while the others do not show such properties (*e.g.* the rhamnolipid purified in this study).

#### 3.5.5 Elemental analysis

Following the outcome of FTIR, 1H NMR and 13C NMR of the extracted biosurfactant, the identity of this compound was then confirmed by elemental analysis where the elemental composition of both crude and purified biosurfactant was investigated (Table 1). Here, the carbon percentage in the crude biosurfactant (before column chromatography) was found as 50.10%, which improved to 61.90% after purification. Concurrently, the proportion of Hydrogen molecule rose 1.3 times higher after purification (9.59%). Meanwhile, in crude sample, the presence of nitrogen and sulphur molecule was evident which was not found after refinement of the product. This suggests the native formation of untreated organic sample.

**Table 1:** Results of elemental analysis of crude and purified biosurfactant

Material	C (%)	O (%)	H (%)	S (%)	N (%)
Crude biosurfactant	50.10	34.64	6.96	5.788	2.51
Purified biosurfactant	61.90	28.57	9.59	—	—

If the synthesized compound is rhamnolipid, (C 26 H 48 O 9, mass = 504.7), then using its molecular mass, we can prove the molecular formula of purified biosurfactant as a monorhamnolipid following to the empirical formula calculation^86^ (Table 2).

**Table 2:** Empirical formula analysis

Component	C (%)	O (%)	H (%)
%	61.90	28.57	9.59
÷Atomic mass	61.90/12	28.57/16	9.59/1.0078
÷Smallest value	5.16/1.79	1.79/1.79	9.523/1.79
Ratio	2.88	1	5.32
Mass of C_2.88_H_5.32_O_1_ = 55.92			
Ratio of mass (504.7/55.92) = 9.02			
So the empirical formula, (C_2.88_H_5.32_O_1_)_×9.02_ = ∼C_26_H_48_O_9_			

#### 3.5.6 The ultraviolet-visible (UV-vis) analysis

**Fig. 9:**
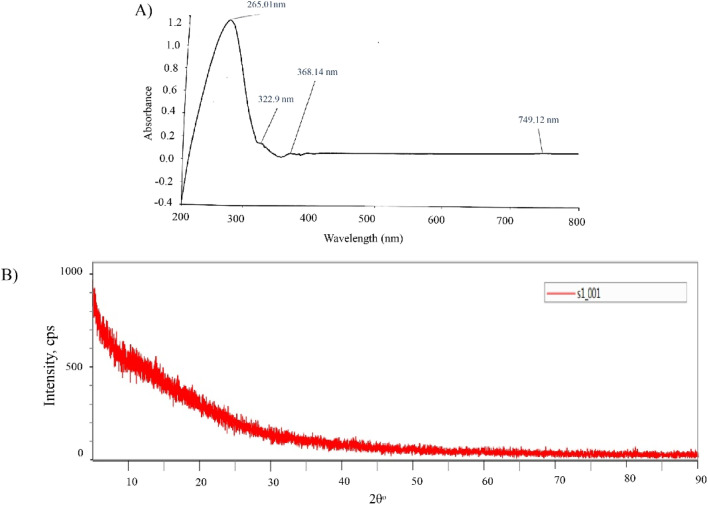
The optical characteristics of purified biosurfactant exhibiting its UV absorbance at 265.01 nm (A), amorphic structure identified by XRD (B).

Fig. 9A shows the optical characteristics of the purified biosurfactant recorded at 200–800 wavelength of UV-vis-NIR. The spectral curve represented a strong absorption edge at wavelength ∼265.01 nm with the occurrence of other absorption peaks at 322.9 and 368.14 nm. These findings coincide with the reports of Schenk and his group where they found similar optical feature exhibited by the extracted rhamnolipid like biosurfactant from *Pseudomonas aeroginosa*.^87^

#### 3.5.7 XRD

X-ray diffraction of the purified biosurfactant exhibited no significant peak except a wide diffracted background (Fig. 9B). This type of appearance suggests the core structure of rhamnolipid as amorphic in nature.^88^

#### 3.5.8 Thermal analysis

**Fig. 10:**
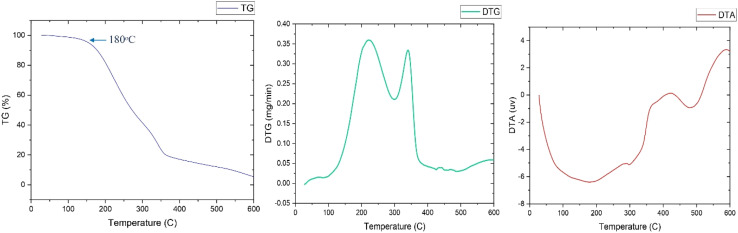
A graphical illustration showing the thermogravimetric (TG), difference thermo-gravimetric (DTG), and differential thermal analysis (DTA) performed on the extracted biosurfactant produced from S2 strain.

The thermostability of the purified biosurfactant is a crucial criterion for its industrial application. From the thermal analysis profiles (Fig. 10), it is clearly evitable that the thermolysis of the sample followed 3 major steps of weight loss between the temperature range of 25 °C and 600 °C. Initially, a gradual loss of 3.7% occurred up to 180 °C due to the release of physically bounded water from the compound, denoting insignificant presence of moisture of the product in that temperature.^89^ A sharp fall in the TG curve from 193 °C to 347 °C revealed the major degradation of the product (approximately 80%), which may associate with the breakdown of rhamnose moiety of rhamnolipid. Abbasi and his colleagues shared a similar observation about rhamnolipid produced by *Pseudomonas aeruginosa*,^90^ where around 80% of the total biosurfactant weight was lost at 300 °C. In the following stage, a decrement of almost 13.4% weight occurred from 374 °C to 596 °C corresponding to the possible decomposition of hydrocarbon chain in the lipid portion. This finding is accompanied by the DTG curve demonstrating maximum weight degradation rate as 360 mg min^−1^ and 334 mg min^−1^ acquired at 223 °C and 344.1 °C respectively. The appearance of a sharp endothermic effect in the DTA curve was evitable from the beginning of the thermolysis process with an extreme at 191 °C attributing to the rhamnose ring degradation. All findings from the thermos-analysis are analogous to the remarks of other studies corresponding to rhamnolipid.^82,91^

As the surfactant isolated in this study was found to be thermally stable up to 180 °C, it can be considered for its commercial application at high temperature reservoir conditions including cleaning.

### 3.6 Determination of foaming properties

**Fig. 11:**
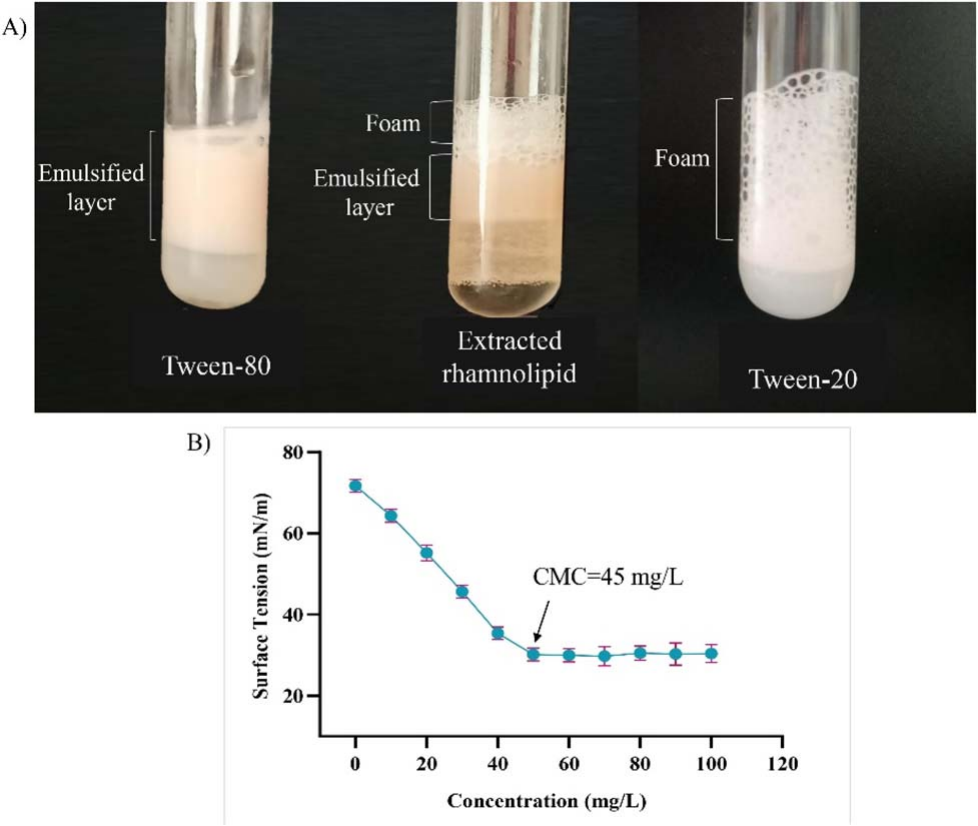
An observation of foaming and emulsification stability of the isolated rhamnolid in presence of pure tween-20 (commercial surfactant) and tween-80 (commercial emulsifier) (A); the CMC of purified biosurfactant determined by plotting the surface tension against the respective concentrations of the surfactant (B). Here, error bars represent the standard deviations (*n* = 3).

Fig. 11A exhibits the remarkable foaming and emulsifying abilities of rhamnolipid biosurfactant (1 g L^−1^) compared to the pure commercial products (tween-20 and tween-80). While tween-20 could only produce foam and tween-80 significantly emulsified the solution; rhamnolipid from this study, showed both characteristics. Several reports on rhamnolipid suggest its notable foaming abilities^92^ while others added up to its emulsification property.^93,94^ Desai *et al.* confirmed that hexose containing fatty acid esters produced by microorganisms are better surfactant than the anionic chemical surfactant as sodium bis(2-ethylhexyl) sulfosuccinate.^49^ Calvo *et al.* reported the significance of rhamnolipid as a bioemulsifier in oil bio-remediation.^95^ In other study, the emulsification property of rhamnolipid was suggested to be used in food industry as bio – preservative.^96^ Therefore, both the emulsification and foaming properties of the isolated rhamnolipid suggests its wider application in different industrial sections.

### 3.7 CMC measurement

As per Fig. 11B, the purified biosurfactant exhibited a CMC of 45 mg L^−1^ showing effective surface activity as it decreased the ST of water from 72 to 30 mN m^−1^. The determination of CMC is quite significant for the industrial use of a surfactant because it denotes the efficacy of the surfactant molecule. As the ST cannot be reduced once the CMC is reached, meaningful use of the surfactant up to a certain amount can be specified.

### 3.8 Antibacterial test

**Fig. 12:**
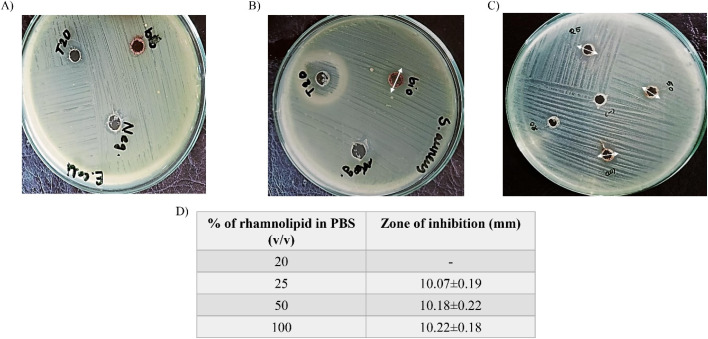
Antibacterial response of rhamnolipid biosurfactant. Antibacterial activity of the biosurfactant in presence of commercial surfactant (tween-20) and negative control (PBS) (labelled as ‘bio’, ‘T20’ and ‘Neg’ respectively) against *E. coli* (A) and *S. aureus* (B) poor inhibition responses were recorded for different concentration of biosurfactants against *S. aureus* (C and D).

In general, rhamnolipids and other biosurfactants are well known for their antibacterial activity.^97,98^ It has been found that rhamnolipid has the permeability to cell membrane, which is why they can inhibit bacterial cell growth to a varying degree.^99^ Several reports have also mentioned their biofilm-disrupting and antiadhesive properties which make them good candidates to be considered as antibacterial agents.^100,101^ In this experiment, antibacterial assay of isolated rhamnolipid was carried out against *S. aureus* and *E. coli* bacterial strains. Based on the inhibition response analysis,^102^ the biosurfactant was found ineffective against *E. coli* while it showed weak activity against *S. aureus* (Fig. 12A and B). Interestingly, the radius of inhibition zones did not extend constantly with the increasing amount of biosurfactant concentrations (25, 50, 100 μg mL^−1^), rather remained same (Fig. 12C). However, Ferreira *et al.*^103^ reported that the antibacterial efficacy of rhamnolipid is pH dependent. The group explained that the Gram-positive pathogens (*S. aureus*) were more sensitive to rhamnolipid at acidic environment while the Gram-negative pathogens (*E. coli*) were resistant to it at all pH studied (pH 5–8). Other reports also suggested mono-rhamnolipid having better antimicrobial applications in agriculture as potential agricultural pathogens, such as *Pantoea agglomerans*, *Alternaria alternata* and *Cladosporium* sp showed significant sensitivity towards it.^104^ It is visible that, the commercial surfactant, tween-20 also showed no activity against the pathogens (Fig. 12A and B). However, there was an opalescent zone around the well containing tween-20 in *S. aureus* lawned plate. This happened due to the lipase production by the bacteria which hydrolyzed tween-20 and produced water insoluble fatty acid precipitation around the well.^105^

### 3.9 Cytotoxicy

**Fig. 13:**
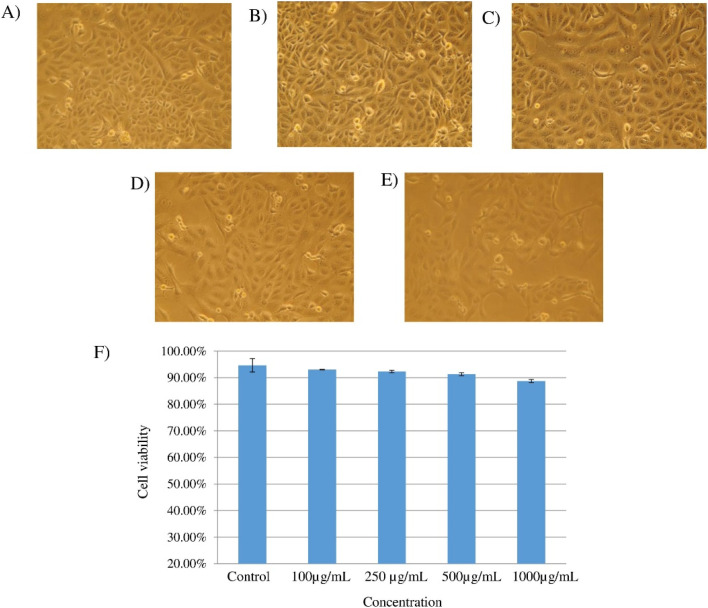
(A–E) microscopic images of the Vero cells – control treatment (A); biosurfactant = 100 μg mL^−1^ (B); biosurfactant = 250 μg mL^−1^ (C); biosurfactant = 500 μg mL^−1^ (D) and biosurfactant = 1000 μg mL^−1^ (E). A bar diagram representing viability rate of Vero cells after treating with biosurfactant in comparison to the control (DMSO) (F). The statistical analysis was performed using SPSS statistical package (version22) for the one-way ANOVA.

Fig. 13A–E depicts the microscopic images of the Vero cells exposed to different concentration of biosurfactant (100, 250, 500, 1000 μg mL^−1^) under controlled conditions, and Fig. 13F shows the corresponding cell viability in comparison to the control. Notably, the viability rate of Vero cells gradually decreased with the increased concentration of biosurfactant treatment but not in a significant way (*p* = 0.10). In comparison to the control (94.63%), the viability rate was found as 93%, 92.33%, 91.20% and 88.70% while treating with 100, 250, 500 and 1000 μg mL^−1^ biosurfactant respectively.

As the isolated biosurfactant was found non-toxic for the epithelial cells (Vero cell line) and showed insignificant antibacterial activity, therefore, both observations suggest the extracted biosurfactant as a safe biomaterial for commercial use as cleaning agent and emulsifier.

## 4. Limitations

The major limitations of this study include the missing of determining cost-effective conditions for large scale biosurfactant production. Also, a broad molecular analysis of the isolate could widen its scope in different industrial use including enzyme and other bio-active compound production. Furthermore, in this study the detailed applications of extracted biosurfactant were not evaluated. All these shortcomings would be considered as prospects for future investigations of the study.

## 5. Conclusion

Rhamnolipid is one of the renowned biosurfactants for being biodegradable and eco-friendly. This makes them better choice for a broad range of applications in food preservation, oil recovery, detergents, cosmetics, and pharmaceuticals. In this present study, a rhamnolipid producer *Bacillus velezensis* S2, isolated from oil-contaminated soil showed remarkable crude oil degradation capacity. When the strain was tested for its biosurfactant producing ability, it confirmed the secretion of a biosurfactant having extensive surface tension reducing and high emulsifying activity. Upon extraction and purification, the biosurfactant product was confirmed as rhamnolipid *via* TLC, NMR, FT-IR and spectrophotometric analysis. The foam test, emulsification index, antibacterial test, cytotoxic analysis, and thermal stability confirmed its safe and wide use in various applications, particularly in cleaning and cosmetics industry. However, the yield amount is considerably poor in proportion to the extraction and purification expenses which largely inhibits its industrial use. A well-defined large-scale fermentation method for an optimum amount of biosurfactant production with minimal down-stream cost demands elaborate studies.

## Data availability

As the corresponding author of this study, I certify that all data underlying the findings are available as the part of the manuscript and no additional source of data is required.

## Author contributions

Shahnaz Sultana: conceptualization, data curation, analysis, methodology, writing original draft, editing. Rokaia Sultana: conceptualization, analysis, methodology, writing review. Md. Abdullah Al-Mansur: data curation, analysis, supervision. Md. Ahedul Akbor: data curation, analysis. Nasrin Akter Bhuiyan: analysis, methodology, writing editing. Shamim Ahmed: investigation, resources. Sabina Yasmin: data analysis. A. H. M. Shofiul Islam Molla Jamal: data analysis.

## Conflicts of interest

The authors declare no conflict of interest.

## Supplementary Material

RA-014-D4RA02572E-s001
