# Supplementary material for: An industrially potent rhamnolipid-like biosurfactant produced from a novel oil-degrading bacterium, Bacillus velezensis S2
Source: RSC Adv. 2024 Aug 5;14(34):24516–33. doi: 10.1039/d4ra02572e (PMC11299641; doi:10.1039/d4ra02572e)
Supplement: RA-014-D4RA02572E-s001 [file RA-014-D4RA02572E-s001.pdf]

Electronic Supplementary Material (ESI) for RSC Advances.
This journal is © The Royal Society of Chemistry 2024

2. Methodology

2.3.2 Phylogenetic analysis by 16S rRNA gene sequences

The isolate was grown in 5 mL of Luria–Bertani medium and the extracted DNA was used as template for polymerase chain reaction (PCR)-based amplification using a PCR Thermal Cycler (Biometra Tone, Analytikjena, Germany). The amplification was carried out using the primer: 27F (5′-AGAGTTTGATCMTGGCTCAG-3′) and 1492R (5′-GGTTACCTTGTTACGACTT-3′) (Chromous Biotech, Bangalore) (Stevens and van Elsas 2010). The PCR program was set as follows:

I. Initial denaturation at 94 °C for 5 min,
II. 30 cycles of Denaturation at 94 °C for 1 min 30 s), annealing at 55 °C for 1 min, and an extension at 72 °C for 1 min 30 s).
III. An additional extension at 72 °C for 5 min

The amplified products were analyzed by gel electrophoresis (PS300TP, Biometra, Germany) in a 1.5% agarose gel with a 100 bp DNA ladder (PROMEGA, USA) and visualized by using an UVP transilluminator (Analytik Jena, US). After conferring the amplification, PCR product was sequenced by an automated DNA sequencer (3500 genetic Analyzer, USA). Full length sequences (1400–1500 bp) were obtained using the Seqman Genome Assembler (DNAstar, USA) by combining the partial sequences, collected from using forward and reverse primers. A phylogenetic tree was constructed using the neighbor-joining method showing the relationship of the isolate with other closely linked reference strains obtained from GenBank database of the National Center for biotechnology Information (NCBI) (http://www.ncbi.nlm.nih.gov/GenBank) using the basic local alignment search tool (BLAST). The Kimura 2-parameter method was used to compute the numbers at nodes that indicate the percentages of 1000 bootstrap (Kimura 1980) in MEGA 6.0 software (Das and Tiwary 2013). Here, *Pseudomonas fluorescens* strain KU 7 (AB266613.1) was used as the outgroup branch. The sequence data of the isolates were deposited onto the GenBank nucleotide sequence database using the BankIt sequence submission under accession number OR337901.

2.4.1 Gravimetric Analysis of crude oil biodegradation

100 mL of n-hexane was taken in a 500 mL separating funnel containing 100 mL of cell free supernatant (CFS) with 10% crude oil and mixed vigorously. The separated organic layer was

eluted and filtered into a 250mL round flask bottle. The procedure was repeated thrice. After extraction, then n-hexane was evaporated in a water bath at 70–75°C. The residue crude oil was then weighed by a digital balance (SHIMADZU, Japan). The quantitative degradation by gravimetric analysis was estimated using the following formula.

$$\% \text{ degradation} = \frac{Weight\ of\ \ degraded\ crude\ oil}{Weight\ of\ residue\ crude\ oil\ in\ control\ medium} \text{ X } 100$$

Here, the weight of degraded crude oil was determined by subtracting the weight of recovered oil from culture media from the weight of the residue oil in the control media.

2.4.2 Spectrophotometric Method

The residual oil present in the culture media was extracted in n-hexane at an interval of seven days up to 28 days of culture incubation following the same procedure described in section 2.4. The amount of extracted residual oil was then recorded by UV spectrophotometer (UV-2600, SHIMADZU, Japan) at *wavelength* of 337 nm using the standard curve for crude oil diluted in n-hexane. To calculate the degraded oil amount in percentage, following equation was used.

% degradation =

(

$$\frac{volume\ of\ residual\ crude\ oil\ in\ control\ medium - volume\ of\ residual\ crude\ \text{[illegible]}}{volume\ of\ residual\ crude\ oil\ in\ control\ medium}$$

)

) X 100

2.4.3 Oil Degradation Analysis by Gas Chromatography (GC)

100 mL of n-hexane was taken in a 500 mL separating funnel containing 100 mL of CFS with 10% crude oil and mixed vigorously. The separated organic layer was eluted and filtered into a 250mL round flask bottle. The procedure was repeated thrice. After extraction, hexane was then evaporated up to 2 mL in a water bath at 70–75 ◦C (Varjani, Rana et al. 2015). The extract containing residue crude oil in n-hexane was then further filtered by a micro-syringe filter (ECOSTAR, HANGHZOU) and collected in GC sample vial for instrumental analysis. The biodegradation of crude oil was examined by injection a 1.0 μL aliquot of final extract into a GC-ECD (Shimadzu 2010 plus) equipped with a 63 Ni electron capture detector and a moving needle-type injection system, where the injection mode was splitless.

The column consisted of SH Rtx-CLP, having a length of 30 mg, an inner diameter of 0.32 mm, and a film thickness of 0.50 μm. Here, the column temperature was started from 120 °C (Habibullah-Al-Mamun, Ahmed et al. 2019) (1 min hold) at a rate of 15°C/min to 200 °C (1 min hold) and then at a rate of 5°C/ min to 300 °C (10 min hold). The temperature of detector and injector was maintained at 300 °C and 200 °C, respectively. Nitrogen was used as the carrier gas with a flow rate of 10.0 $mLmin^{-1}$. The chromatograms were analyzed with GCsolution Postrun (Version 2.41.00) with a workstation (GCsolution), and a computer for data acquisition and processing.

2.5.1 Oil Spreading Assay (OSA)

40 mL of distilled water (Milli-Q) in a Petri plate with diameter of 20 cm was taken. Then 100 μL of crude oil was dropped onto the surface of the water, followed by the addition of 20 μL of CFS on the center of the thin oil layer. CFS was collected from an overnight incubated culture ($1.2*10^8$ CFU/mL) grown in MSM and 10% oil containing media for 24 h and 37◦C. After the placement of CFS, if the oil is displaced and a clear zone is formed on the oil-water interface, the diameter of the clear zone was measured and compared to 20 μL of distilled water as a negative control (Mouafi, Elsoud et al. 2016) and 20 μL of Tween 20 and SDS were used as positive controls.

2.5.2 Drop collapse test (DCT)

2 μl of crude oil was applied to 96- well microtiter plates. 5μl of CFS was added to the surface of the oil in the well followed by the equilibration of the plate for 1 h at 37°C. The drop shape was being observed for 1 min using a magnifying glass. The result was considered positive for biosurfactant production when the drop collapsed and if the drops remained intact or round, they scored as negative indicating the lack of biosurfactant production. In this experiment, distilled water was used as negative control treatment, while Tween 20 and SDS were used as positive control (Korayem, Abdelhafez et al. 2015).

2.5.3 Emulsification Index (E_{24}):

The test was performed by mixing two milliliters of crude oil (2 mL) with 2 mL of CFS in a test tube and vortexing vigorously at high speed for 2 min. The mixtures were left to stand for 24 h, and the emulsion index (E_{24}) was calculated as indicated in the following formula:

E24 (%) = total height of the emulsified layer/total height of the liquid layer × 100.

Here, Tween 80 and PBS were used as positive and negative controls respectively.

2.5.4 Bacterial adhesion to hydrocarbon (BATH) assay

The cells were first washed couple of times to remove any interrupting solutes and then resuspended in a buffer salts solution (pH 7.0) containing 16.9 g of K_2HPO_4, 7.3 g of KH_2PO_4, 0.2 g of $MgSO_4$.$7H_20$, and 1.8 g of urea per liter to give an optical density (OD) of ~ 0.5 at 610 nm. Following that, 1 mL of crude oil was added to the 4 mL of cell suspension in a screw-cap test tube (18ml, 16x150mm) and the test tube was vortex-shaken for 3 min. After shaking, aqueous phases and crude oil were allowed to separate for 1 hr. After careful removal of aqueous phase with a Pasteur pipette, the OD of the aqueous phase was then measured at 610 nm. The hydrophobicity of cells is expressed as the percentage of their adherence to crude oil, which is calculated as follows:

$$\% \text{ of bacterial cell adherence} = 1\text{-}\left(\frac{OD\ of\ the\ aqueous\ phase}{OD\ of\ the\ cell\ suspension}\right) \text{ x } 100$$

2.5.5 Surface Tension Determination

The CFSs were collected from five different conc. of grown culture ($1.3*10^8$, $3.2*10^8$, $8.0*10^8$, $2*10^9$ and $2*10^3$) with OD value of 0.2, 0.4, 0.6, 0.8 and 0.98 respectively. The syringe was blunt shaped and had a needle of 0.47 mm diameter. Prior to measurement, the glass slides were cleaned thoroughly with deionized water, dried, and then placed. Each droplet excreted from syringe was 5 µL with each measurement repeating three times. Here Deionized (DI) water and sterile nutrient broth was used as a control (Nitschke, Ferraz et al. 2004).

2.6 Biosurfactant extraction:

Same media composition of MSM (250 mL) supplemented with 1% glucose and 10% crude oil was prepared. The media was then inoculated with 1 ml of fresh culture (3.5×10^8 CFU/mL) of the respective isolate and incubated at 30°C, 120 rpm for 7 days. The cells were then removed by centrifugation (Model-DSC-200A-2, DigiSystem, Taiwan) at 6000 rpm and 4°C for 20 minutes. The supernatant was taken, and the pH of the supernatant was adjusted to 2, using 6N HCl. Then equals volume of chloroform: methanol (2:1) was added for liquid-liquid extraction of biosurfactant (Goyal and Singh 2022).The mixture of supernatant and organic solvent was shaken vigorously and after the stable separating layer took place, the organic layer (light amber color) containing the crude biosurfactant was collected (Anandaraj and Thivakaran 2010, Tripathi, Gaur et al. 2020). The organic solvent was evaporated using a rotary evaporator (BUCHI, Switzerland) and the honey-colored residue was the unpurified biosurfactant (Saravanan and Vijayakumar 2012). Similar procedure was followed for the extraction of biosurfactant with equal volume of ethyl acetate as organic solvent to evaluate which solvent works better for biosurfactant extraction.

2.7 Purification of biosurfactant and thin layer chromatography

A bit of cotton was wedged into the bottom of the burette and the burette was filled with silica to 40-45 inches high. Briefly, hexane was added to the column silica which made a gel like appearance. Cotton soaked in hexane was placed on the top of the silica-hexane gel (worked as solid phase) on which the sample was placed. Then, hexane; 90% hexane: 10% ethyl acetate; 50% hexane: 50% ethyl acetate; 100% ethyl acetate; 50% ethyl acetate: 50% methanol and finally methanol were added periodically as mobile phase (Cooper and Goldenberg 1987). The eluents from the column were collected sequentially in clean test tubes and the solvents were allowed to evaporate using rotary evaporator leaving the fractionated compounds.

A thin layer chromatography (TLC) was performed to check the purity of the fraction where chloroform–methanol water (65:15:2, v/v) solvent system was used as mobile phase.

2.8.3 Rhamnose detection

A volume of 0.5 mL of cell supernatant ($2.1*10^5$ CFU/mL) after 24 hour of incubation was mixed with 0.5 mL of 5% phenol solution and 2.5 mL of sulfuric acid. The mixture was incubated for 15 min before measuring absorbance at 490 nm. The baseline of the instrument was constructed using deionized water.

2.8.7 Thermal properties analyses

3.5 mg of biosurfactant sample was loaded in the alumina sample holder and heated over a temperature range of 25–600°C at a constant temperature gradient of 20°C/min under nitrogen atmosphere. To determine the decomposition temperature of biosurfactant, Thermal Gravimetric (TG) Analysis was carried out. As well, from the results of TGA analysis, the Difference Thermo-gravimetric (DTG) curves were also drawn which indicated the temperatures where the maximum or minimum rate of weight was lost for the sample. Also, the Differential Thermal Analysis (DTA) curve generated from the experiment gives melting and degradation temperatures of the samples.

2.8.8 Determination of foaming property:

1 g/L of extracted biosurfactant was taken into each test tube (a set of three) followed by the addition of distilled water and crude oil (1 mL each). Here, tween-20 (1 mL) and tween-80 (1 mL) were used as positive controls for commercial surfactant and emulsifier respectively. Each tube was hand shaken vigorously for several minutes and left undisturbed for 2 h. The stability of the produced foam and emulsification was observed thereby.

2.8.10 Antibacterial test

Mueller-Hinton agar plates were prepared where around 1.2×10^{8} CFU/mL of each culture were swabbed over the solidified agar. The McFarland Standard Formula (Trenholme, Kaplan et al. 1989) was prepared to confirm the inoculum growth concentration and the bacteria were enumerated by the conventional plate count method (Taylor, Allen et al. 1983). Subsequently, a sterile cork-borer was used for making three wells (6 mm diameter, 5 mm in depth) in the solidified agar; where one well was used for biosurfactant and other two wells were used for Tween-20 and sterile phosphate buffer saline (PBS) as a commercial surfactant and negative control respectively. Simultaneously, various concentrations (20%, 25%, 50%, and 100 %) of 30μL biosurfactant in PBS (v/v) were prepared, sterilized by syringe filter (0.22 μm) and and poured into separate wells punctured in *S.aureus* and *E.coli* lawned MH agar plates. The plates were incubated at 37°C for 24 hours and the clear zone diameters around the well were observed (Saravanakumari and Mani 2010).

2.8.11 Cell cytotoxicity

The cell line selected for this experiment was the Vero cell line (CLS 605372, Germany) established from the Kidney epithelial cells isolated from African Green Monkey. Prior to the cytotoxic experiment, biosurfactant was taken into DMSO to prepare a concentration range of 100, 250, 500 and 1000 $mgmL^{-1}$. Then the cell line was treated with the respective concentrations of the compound and each set of experiments was performed thrice by applying afresh treatment doses. After culturing for 24 hours, cell suspension (1 mL) from each treatment was taken for cell counting (both viable and non-viable). An automated cell counter (LUNA-II™, Analytikjena) was used to determine the viable (unstained) and non-viable (stained) cells following the trypan Blue Exclusion method. The cell viability (%) was calculated as follows:

$$\% \text{ of cell viability} = \left(\frac{number\ of\ viable\ cells}{total\ number\ of\ cells}\right) \times 100$$

Here, only DMSO was used as a control treatment to compare the observed results.
